# Adult Vaccine Coadministration Is Safe, Effective, and Acceptable: Results of a Survey of the Literature

**DOI:** 10.1111/irv.70090

**Published:** 2025-03-05

**Authors:** Litjen Tan, Dana Trevas, Ann R. Falsey

**Affiliations:** ^1^ Immunize.org Saint Paul Minnesota USA; ^2^ Shea and Trevas, Inc Brandywine Maryland USA; ^3^ University of Rochester Medical Center Rochester New York USA

**Keywords:** COVID‐19 vaccines, immunization coverage, influenza vaccines, public health, RSV vaccines, vaccination, vaccine administration, vaccine combinations, vaccine‐preventable diseases

## Abstract

**Background:**

Coadministration of vaccines in children is a long‐standing practice that has proven to be safe and effective in improving the efficiency of vaccine administration, thereby increasing immunization coverage rates. As the number of vaccines routinely recommended for adults increases, and with limited opportunities for adults to have preventive health touchpoints with providers, adult vaccine coadministration should be considered as a routine practice to improve vaccination coverage rates and public health. A review of existing literature was conducted to examine the potential reactogenicity and impact on effectiveness when co‐administering vaccines to adults.

**Methods:**

Medline was searched for research articles with the search term “influenza vaccine” or “vaccination,” combined with the search terms “simultaneous,” “concomitant,” “concurrent,” and “combination.” Another search of Medline was conducted on the search term “influenza vaccine” or “vaccination” combined with the following individual search terms: “RSV,” “COVID,” and “Tdap.” The references of extracted articles were also examined for potential other relevant articles.

**Results and Conclusions:**

Adult vaccine coadministration is safe for all the combinations we assessed. Most adverse events (AEs) were generally mild to moderate and of short duration. Some studies showed slightly more reactogenicity with coadministration but few or no serious AEs or safety signals. Nearly every study confirmed that coadministration had no significant effect on immune response for either vaccine. The benefits of vaccine coadministration outweigh the risks. It increases convenience for vaccinees, reduces the number of missed opportunities to vaccinate, and contributes to efficient use of healthcare resources.

## 1 Introduction

Coadministration of vaccines—also called concomitant or simultaneous vaccination—in children is a long‐standing practice that has proven to be safe and effective in improving the efficiency of vaccine administration, thereby increasing immunization coverage rates. In 2021, as COVID‐19 vaccines became available and questions arose about their interaction with seasonal influenza vaccines, the World Health Organization (WHO) reviewed unpublished data to assess the benefits and risks of coadministration of these two vaccines in adults. Because of concerns about the reactogenicity of some COVID‐19 vaccines when given alone, WHO paid particular attention to reactogenicity. WHO concluded that coadministration does not interfere with the effectiveness of either the COVID‐19 or influenza vaccine, that the amount of reactogenicity is acceptable, and coadministration is tolerable for vaccinees [1]. Since then, more research has been conducted, and the preliminary findings reviewed by WHO have been published, supporting coadministration of COVID‐19 and seasonal influenza vaccines. Moreover, the Centers for Disease Control and Prevention (CDC) and the WHO concur that coadministration of most types of vaccines is acceptable [2].

For decades, adults have received multiple vaccines in preparation for international travel, demonstrating that coadministration can be an effective tool in vaccinating adults. As the number of vaccines routinely recommended for adults increases, and with limited opportunities for adults to have preventive health touchpoints with providers, adult vaccine coadministration as a routine practice is a growing consideration for protecting against vaccine‐preventable diseases and improving public health. We conducted a review of existing literature to examine the potential reactogenicity and impact on effectiveness when co‐administering vaccines to adults. Our review of recent studies of coadministration of seasonal influenza vaccines with either COVID‐19 or respiratory syncytial virus (RSV) vaccines suggests that the approach is safe, effective, and acceptable to most people. We also highlight studies supporting the safety and effectiveness of other vaccine combinations in adults.

## 2 Methods

To develop this narrative review, we searched Medline for research articles with the search term “influenza vaccine” or “vaccination,” combined with the search terms “simultaneous,” “concomitant,” “concurrent,” and “combination.” Another search of Medline was conducted on the search term “influenza vaccine” or “vaccination” combined with the following individual search terms: “RSV,” “COVID,” and “Tdap.” The references of extracted articles were also examined for potential other relevant articles. All articles were examined, and any article that was not directly related to coadministration of vaccines was excluded.

## 3 Results

### 3.1 Influenza Plus COVID

Table 1 outlines the findings of published clinical trials on coadministration of COVID‐19 and influenza vaccines.

**TABLE 1:** Summary of published studies of COVID‐19 plus influenza vaccine coadministration.

Author	Design	Population and study groups	Immunogenicity	Safety and reactogenicity	Conclusion
Lazarus et al. [3]	Randomized, controlled	Adults 18 years and older who received one (initial) dose of COVID‐19 vaccine (ChAdOx1 or BNT162b2) and received a second dose with or without an age‐appropriate influenza vaccine	Immune responses were not adversely affected.	No safety concerns Coadministration is noninferior compared with the COVID‐19 vaccine alone in terms of any systemic AE within 7 days.	Coadministration raised no safety concerns, produced acceptable reactogenicity profiles, and preserved binding antibody responses.
Toback et al. [4]	Randomized, controlled, open‐label	Adults 18–64 years old received an initial dose of the COVID‐19 vaccine (NVX‐CoV2373) plus an age‐appropriate influenza vaccine or a placebo plus an age‐appropriate influenza vaccine.	Modest decrease in Novavax immunogenicity with coadministration (possibly attributable to coadministration with the initial dose of the COVID vaccine), but no interference with the influenza vaccine immune response.	No safety concerns Coadministration had no clinically meaningful effect on systemic or local reactogenicity.	Coadministration is safe, immunogenic, and effective.
Izikson et al. [5]	Randomized, controlled, open‐label	Adults 65+ years received either the COVID‐19 vaccine (mRNA‐1273) or the high‐dose quadrivalent influenza vaccine or both vaccines concomitantly.	No immune interference with coadministration	No safety concerns	Coadministration is safe and immunogenic.
Radner et al. [6]	Prospective, open‐label, cohort	Adults 18 years and older received the COVID‐19 vaccine (BNT162b2) booster only, the influenza vaccine only, or a combination of both vaccines concomitantly.	Four weeks after COVID‐19 booster dose, significantly higher median anti‐RBD‐antibody levels in the booster‐only arm than the combination arm; clinical relevance unclear	No safety concerns Frequency and severity of AEs are similar in the booster‐only and combination arms but low in the influenza‐only arm.	Coadministration shows no safety concerns but may reduce the immunogenicity of the COVID‐19 vaccine.
Naficy et al. [7]	Randomized, open‐label	Adults 18 years and older received the COVID‐19 vaccine (mRNA‐1273) booster and influenza vaccine concomitantly or sequentially (2 weeks apart).	No immune interference in either group	No safety concerns	Coadministration was immunologically non‐inferior to sequential administration, and both groups had comparable safety and reactogenicity profiles.
Gonen et al. [8]	Prospective, cohort	Adults 18 years and older received the COVID‐19 vaccine (Omicron BA.4/BA.5–adapted bivalent) or influenza vaccine or both concomitantly.	Coadministration resulted in a mild decrease in anti‐spike immunoglobulin‐G titers, but no COVID‐19 infections were reported	Coadministration reactogenicity similar to that of COVID‐19 vaccine alone	Coadministration not associated with substantially inferior immune response or more frequent adverse events than COVID‐19 vaccine administration alone
Choi et al. [9]	Open‐label, non‐randomized	Adults 18 years and older received either a concomitant bivalent COVID‐19 mRNA booster and influenza vaccination concomitantly or sequentially (at least 4 weeks apart).	Coadministration induces sufficient immunogenicity, but potential immune interference cannot be ruled out. Coadministration of multiple heterologous antigens might attenuate the immune imprinting phenomenon.	No safety concerns No clinically relevant increase in AEs with coadministration	Coadministration is sufficiently immunogenic and has a tolerable safety profile. Depending on the previously exposed vaccine antigens and the similarity or number of coadministered vaccine antigens, immune responses might be variably induced through immune imprinting and interference.
Hause et al. [10]	Retrospective, cohort	People aged 12 years and older had the COVID‐19 mRNA booster vaccine alone or with the influenza vaccine and volunteered to respond to health surveys through V‐SAFE, a smartphone‐based active safety surveillance system.	n/a	No serious safety concerns Coadministration associated with significant increases in reports of systemic reactions up to 7 days following vaccination	Coadministration is safe but may result in slightly more systemic reactions in the week following vaccination than COVID‐19 booster alone.
McGrath et al. [11]	Retrospective, comparative effectiveness	Adults 18 years and older received the COVID‐19 vaccine (BNT162b2 bivalent) booster only, the age‐appropriate influenza vaccine only, or a combination of both vaccines concomitantly.	No immune interference	Slight differences in COVID‐related outcomes in the coadministration group were minimized after adjusting for negative control outcomes.	Coadministration is safe and associated with generally similar effectiveness in the community setting than giving either vaccine alone.
Janssen et al. [12]	Systematic review	Review of published data on the safety, immunogenicity, efficacy/effectiveness, and acceptability/acceptance of coadministration of influenza and COVID‐19 vaccines in adults 18 years and older.	No immune interference One study (Toback et al.) showed a modest decrease in NVX‐COV2373 immunogenicity with coadministration.	No safety concerns Similar reactogenicity profiles between coadministration and COVID‐19 vaccine‐only groups and lower in the influenza vaccine group than in the coadministration and COVID‐19 vaccine‐only groups	Coadministration is safe and does not induce immunological interference; it appears to be a good strategy to protect individuals in a timely manner against both infections and their complications and to reduce stress on healthcare systems.
Moro et al. [13]	Database Review	Assessment of reports to VAERS database following mRNA COVID‐19 vaccination plus influenza vaccination or first booster dose of COVID‐19 vaccine alone in people of all ages	n/a	No new or unexpected safety issues Systemic reactions are more frequent with coadministration than with the COVID booster vaccine alone.	No new or unexpected safety issues were identified for coadministration when compared with giving either vaccine alone.
Pattinson et al. [14]	Longitudinal, cohort	Assessment of blood and serum samples from people of all ages who received influenza vaccine and bivalent COVID‐19 vaccines concomitantly, either ipsilaterally or contralaterally	Response to SARS‐CoV‐2 was slightly increased in the ipsilateral group, but equivalence was not excluded.	n/a	Coadministration in the same arm or different arms did not strongly influence the antibody response to either vaccine.

Abbreviations: AE, adverse event; n/a, not applicable; RBD, receptor binding domain; SARS‐CoV‐2, severe acute respiratory syndrome coronavirus‐2; VAERS, Vaccine Adverse Event Reporting System.

#### 3.1.1 Safety

None of the studies reviewed found serious safety concerns with coadministration of COVID‐19 and influenza vaccines [3, 4, 5, 6, 7, 8, 9, 10, 13]. All of the randomized, controlled trials (RCTs) [3, 4, 5, 7] showed that adverse events (AEs) were few and balanced across groups. Janssen et al. determined that most AEs were mild to moderate and self‐limiting. Janssen et al. also highlighted the absence of safety alerts in countries that implemented coadministration for the 2021–2022 influenza season [12]. Moro et al.'s review of the Vaccine Adverse Event Reporting System (VAERS) from 2021 to 2022 confirmed there were no unusual or unexpected patterns of AEs [13].

#### 3.1.2 Reactogenicity

All of the RCTs determined that coadministration had acceptable reactogenicity [3, 4, 5, 7]. The most common reported events were mild‐to‐moderate injection site pain [4, 7] and mild‐to‐moderate fatigue, headache, and myalgia [3, 4]. Naficy et al. found that injection site pain was more frequent in the coadministration group [7]. Others found lower reactogenicity and fewer AEs in influenza‐vaccine‐only groups [5, 6, 12]. Toback et al. found reactogenicity was more common with coadministration than with the COVID‐19 vaccine alone, a finding supported by Moro et al.'s review of the VAERS database [4, 13].

Hause et al.'s assessment of CDC's V‐SAFE, a smartphone‐based, active safety surveillance system, revealed that coadministration was associated with significant increases in reports of systemic reactions within the week following vaccination compared with mRNA COVID‐19 vaccine alone [10]. Specifically, coadministration of influenza vaccine with the Pfizer vaccine increased reports by 8% and with the Moderna vaccine, 11%. The study authors suggested these small increases could have resulted from residual confounding between those who received COVID‐19 vaccination simultaneously with influenza vaccine versus those who only received a COVID‐19 booster and likely do not have clinical significance.

In their comparative effectiveness study of more than 3 million adults enrolled in either commercial insurance or Medicare Advantage plans, McGrath et al. found a similar effectiveness in both the coadministration group and the COVID‐19–vaccine‐only group [11]. Their unadjusted results showed slightly more COVID‐related outcomes in the coadministration group than the COVID‐19–vaccine‐only group but a slightly lower incidence of influenza‐related outcomes compared with those who received the influenza vaccine only. These differences were minimized after adjusting for negative control outcomes (i.e., urinary tract infection and unintentional injury). The study authors said that the differences were likely explained by residual bias between vaccine exposure groups.

#### 3.1.3 Immunogenicity

Most studies found that coadministration did not result in immunologic interference [3, 5, 7, 11, 12]. Toback found a modest decrease in the immunogenicity of the Novavax COVID‐19 vaccine when coadministered with the influenza vaccine (possibly attributable to the non‐randomized nature of the studied groups) but no evidence of interference with the influenza vaccine [4]. Gonen et al. concluded that coadministration resulted in a mild decrease in anti‐spike immunoglobulin‐G titers, but no COVID‐19 infections were reported, and the immune response with coadministration was not substantially inferior to that of COVID‐19 vaccination alone [8]. Pattinson et al. determined that coadministration of influenza and bivalent COVID‐19 vaccines in the same arm or different arms did not strongly influence the antibody response to either vaccine [14].

Choi et al.'s open‐label, non‐randomized study found that immune response against Omicron BA.5 was higher in the coadministration group than the sequential administration group; in contrast, coadministration was inferior to sequential administration against the wild‐type (or ancestral) SARS‐CoV‐2 strain of COVID‐19 [9]. The study authors hypothesized that coadministration with multiple heterologous antigens might affect immune imprinting. They added that the ancestral strain accounts for an extremely small proportion of currently circulating COVID‐19 virus. Choi et al. concluded that coadministration induces sufficient immunogenicity, but potential interference could not be ruled out.

Radner et al.'s prospective, open‐label, cohort study determined that coadministration, specifically with the Pfizer COVID‐19 booster vaccine, resulted in reduced immunogenicity compared with the COVID booster vaccine only [6]. They suggested that lower immunogenicity could result in more breakthrough infections but concluded that the clinical relevance of their finding requires further evaluation.

#### 3.1.4 Uptake and Acceptability

Through a retrospective, cross‐sectional study of Medicare data, Harris et al. determined that Medicare beneficiaries had high uptake of the influenza vaccine but low rates of coadministration with the COVID vaccine (although coadministration rates in the study population increased from 11.1% in the fall of 2021 to 36.5% in the fall of 2022) [15]. Medicare beneficiaries who had dementia or lived in rural counties were more likely to have coadministration than other subpopulations. Harris et al. suggested that some of the racial and ethnic differences detected in rates of coadministration may be more related to access and awareness than acceptance.

Parker et al. used CDC's V‐SAFE platform to assess trends in coadministration of COVID‐19 vaccine with other vaccines, almost entirely in adults (more than 97%), of whom 85% received influenza vaccine along with COVID‐19 vaccine [16]. They concluded that coadministration of COVID‐19 vaccine with any other vaccine increased from 9.2% in the 2021–2022 influenza season to 34.4% in the 2022–2023 influenza season, possibly reflecting increased acceptance.

Houle et al.'s survey of Canadians' knowledge, attitudes, and beliefs about coadministration, conducted during the 2022–2023 influenza season, revealed that 70% were aware of the option of coadministration but only 26% received coadministration—most often related to vaccine availability and scheduling [17]. The survey found that 22% of those who were aware were not interested in coadministration. Of these, 20% did not see a benefit to coadministration, and 17% were concerned about compounded adverse effects. The survey saw higher rates of awareness regarding vaccine coadministration among people at high risk, people age 65 years and older, and people who had previously received either vaccine.

### 3.2 Influenza Plus RSV

Table 2 summarizes the published studies of coadministration of influenza and RSV vaccine. Our review looked at six RCTs and one systematic review.

**TABLE 2:** Summary of published studies of influenza plus RSV vaccine coadministration.

Author	Design	Population and study groups	Immunogenicity	Safety and reactogenicity	Conclusion
Falsey et al. [18]	Randomized, controlled	Adults 18–49 and 65–85 years received RSVpreF vaccine or placebo alone or concomitantly with influenza vaccine.	Coadministration had no substantive impact on immunogenicity. Influenza vaccine responses trended lower with coadministration and were more pronounced among younger adults.	No safety concerns	Coadministration is well tolerated and highly immunogenic in older adults.
Chandler et al. [19]	Randomized, controlled, open‐label	Adults 60 years and older received the adjuvanted RSVPreF3 older adult vaccine and influenza vaccine concomitantly or sequentially (30 days apart).	Coadministration is noninferior to sequential administration.	No safety concerns	Coadministration well tolerated, with an acceptable safety profile.
Buynak et al. [20]	Randomized, controlled, open‐label	Adults 65 years and older received the adjuvanted RSVpreF3 older adult vaccine and influenza vaccine concomitantly or sequentially (1 month apart).	Coadministration is noninferior to sequential administration.	No safety concerns Slight increase in systemic events with coadministration, but no clinically meaningful differences between groups.	Coadministration in older adults is immunogenically noninferior to sequential administration, well tolerated, with an acceptable safety and reactogenicity profile.
Clark et al. [21]	Randomized, open‐label	Adults 65years and older received the RSVpreF3 older adult vaccine and influenza vaccine concomitantly or sequentially (1 month apart).	Coadministration is noninferior to sequential administration. No immune interference	No safety concerns More systemic events after coadministration, but not clinically relevant	Coadministration in older adults is immunogenically noninferior to sequential administration, with an acceptable safety and reactogenicity profile.
Baber et al. [22]	Randomized, controlled, observer‐blind	Adults 65–85 years received RSVpreF (adjuvanted or unadjuvanted) vaccine or placebo concomitantly with influenza vaccine	Unadjuvanted RSVpreF induced a robust response; adjuvanted formulations did not enhance the response.	No safety concerns	Coadministration well tolerated, with an acceptable safety profile.
Athan et al. [23]	Randomized, double‐blind, controlled	Adults 65 years and older received the bivalent RSVpreF vaccine concomitantly with the influenza vaccine or either vaccine alone.	Coadministration is noninferior to either vaccine alone. Influenza vaccine responses are similar or slightly lower with coadministration.	No safety concerns	Coadministration in older adults is immunogenically noninferior to either vaccine alone, with an acceptable safety and tolerability profile; results support coadministration in adults 65 years and older.
Melgar et al. [24]	Systematic review	Review of available evidence on the safety, immunogenicity, and efficacy of influenza and RSV vaccines among adults aged 60 years and older	Coadministration is noninferior for immunogenicity except against the FluA/Darwin H3N2 strain (with the GSK RSV vaccine plus adjuvanted quadrivalent inactivated influenza vaccine). Antibody titers lower with coadministration; clinical significance is unknown.	Mixed evidence on coadministration reactogenicity	Consider patient factors in the decision for coadministration.

Abbreviation: RSVpreF, respiratory syncytial virus prefusion F vaccine.

#### 3.2.1 Safety

None of the studies reviewed identified safety concerns with coadministration of influenza and RSV vaccines [18, 19, 20, 21, 22, 23, 24]. All of the clinical trials found coadministration was well tolerated [18, 19, 20, 21, 22, 23]. AEs were similar across RSVpreF groups [18]. No serious AEs were considered related to the study vaccines [18, 23]. No clinically meaningful differences in local or systemic solicited and unsolicited AEs were identified [20, 21].

#### 3.2.2 Reactogenicity

The trials found that people who had coadministered vaccines experienced mostly mild‐to‐moderate local and systemic AEs of short duration [19, 21, 23], with a low number of Grade 3 events [19]. The observed unsolicited AEs, serious AEs, and potential immune‐mediated disorders were balanced between the groups, and no clustering of events or safety concerns was identified [19].

Clark et al. determined that solicited systemic AEs were reported more frequently after coadministration than after each vaccine separately [21]. However, this finding was not considered clinically relevant, as the coadministration group demonstrated no increase in severity or duration of these events.

Baber et al. found that most systemic and local reactions were mild and occurred more frequently after the RSVpreF vaccine than placebo [22]. Most local reactions (e.g., injection‐site pain, redness, and swelling) were transient and of short duration.

#### 3.2.3 Immunogenicity

The studies we reviewed agreed that, for all ages, coadministration of the RSVpreF vaccine with the influenza vaccine did not affect RSV immunogenicity, regardless of dose level or formulation [18, 19, 20, 21, 22, 23, 24]. Athan et al. found that the response to the influenza vaccine alone was similar or slightly lower with coadministration of the RSVpreF vaccine, with hemagglutination inhibition (HAI) titers for H3N2 trending lower than the other influenza strains tested but meeting noninferiority criteria. The authors did not address the clinical relevance of this finding [23]. Melgar et al.'s systematic review found RSV and influenza antibody titers were lower with coadministration, but the clinical significance of this finding is unknown [24]. Melgar et al. also concluded that coadministration with the GSK RSV vaccine did not meet noninferior criteria in the case of FluA/Darwin H3N2 strain [24].

Falsey et al.'s dose‐finding study demonstrated that coadministration of RSVpreF and influenza vaccines did not affect RSV immunogenicity, regardless of dose level or formulation [18]. However, as seen with the study by Athan et al., immune responses to influenza vaccines trended lower with coadministration than with influenza vaccine alone. The reduced immune response to the influenza vaccine was more pronounced in younger adults than in older adults, who received a high‐dose influenza vaccine. The authors indicated that the reason for the interference is unclear but noted that RSVpreF is a relatively immunodominant antigen.

### 3.3 Influenza Plus Other Vaccines

An RCT by Schwarz et al. showed that coadministration of herpes zoster and influenza vaccines in adults aged 50 years and older did not interfere with the immune response of either vaccine and yielded no safety concerns [25]. Some general reactions were more frequent with coadministration. Overall, local and general reactogenicity was higher for the herpes zoster vaccine than for the influenza vaccine. After the second dose of herpes zoster vaccine, local and general reaction incidences were similar in the two groups.

Cannon et al.'s randomized, double‐blind study of the 20‐valent pneumococcal conjugate vaccine (PCV20) plus influenza vaccine in adults 65 and older found coadministration resulted in noninferior immune responses for all 20 pneumococcal serotypes and all four influenza vaccine strains, compared with either vaccine alone [26]. The safety profile of PCV20 was similar with and without influenza vaccine. Local reactions and systemic events were mostly mild or moderate in severity across groups. Mild and moderate fatigue were reported more frequently after coadministration compared with separate administration, but the findings were not considered clinically significant. The rates of reported AEs were similar across groups, and no serious AEs were considered vaccine‐related.

Chen et al. conducted a prospective trial to assess the coadministration of COVID‐19, influenza, and the combination tetanus, diphtheria, and acellular pertussis (Tdap) vaccines (in various combinations) in pregnant women [27]. They found no safety issues and no negative interactions among the vaccines. Notably, the authors determined that receiving at least three doses of the COVID‐19 vaccine, even without the influenza vaccine, maintained protection against influenza A (but not influenza B) in pregnant persons. While the authors did not provide a mechanism for this indirect protection against influenza A, they emphasize that continued influenza vaccination for pregnant women is essential, particularly because there was no similar protection seen for influenza B.

### 3.4 Other Vaccine Combinations

In their RCT, Hermida et al. found no safety concerns with coadministration of RSV prefusion (RSVpreF3) vaccine plus the diphtheria, tetanus, and acellular pertussis (dTpa) combination vaccine in healthy, nonpregnant women ages 18–45 years [28]. In this study, coadministration yielded a robust immune response to RSVpreF3. However, there was a lower immune response to dTpa when coadministered with RSVPreF3. The authors determined that this finding had no clinical relevance for diphtheria and tetanus, but the clinical significance for pertussis is unclear. Hermida et al. concluded that coadministration is safe for healthy, nonpregnant women ages 45 and younger.

Reikkinen et al. looked at the coadministration of the hepatitis A vaccine—the most common travelers' vaccine—and the 13‐valent pneumococcal conjugate vaccine (PCV13) in adults and found no safety concerns and no immune interference with PCV13 (coadministration elevated immune responses to serotype 3, but that should not greatly affect protection, as overall absolute responses remained low in both groups) [29]. However, there is one clear exception to the effectiveness of adult vaccine coadministration in the study. PCV13 vaccine coadministration interferes with hepatitis A vaccine response. They noted that the findings align with other studies showing immune interference with coadministration of glycoprotein vaccines. Reikkinen et al. called for further research to determine whether the differences in hepatitis A response with coadministration persist with longer follow‐up and whether giving a second dose of hepatitis A vaccine without PCV13 vaccine could compensate for the insufficient response. The study authors concluded that coadministration might not provide sufficient protection against hepatitis A and raised concerns about the effectiveness of coadministration with only one dose of hepatitis A vaccine (a common approach for short‐term protection when travel is imminent).

Ali et al. reviewed five RCTs, each looking at the coadministration of recombinant zoster virus (RZV, or shingles) vaccine with another common adult vaccine: quadrivalent influenza, PCV13, mRNA COVID‐19, Tdap, or 23‐valent pneumococcal polysaccharide vaccine (PPSV23) [30]. All the trials compared coadministration with sequential administration in adults 50 years or older and found similar vaccine response rates regardless of the timing. (One pertussis antigen, pertactin, did not meet the criteria for noninferiority with coadministration, but the study authors noted that other vaccine coadministration studies have come to the same finding, and the clinical relevance is unknown.) All five studies found similar rates of AEs between coadministration and sequential administration groups. Ali et al. concluded that coadministering the RZV vaccine with other routine adult vaccines does not significantly affect the reactogenicity, immunogenicity, or safety of the RZV vaccine or the coadministered vaccine [30].

Melgar's systematic review concluded that data are lacking on the safety of coadministration of RSV vaccine with other vaccines that might be recommended for people ages 60 years and older, such as COVID‐19, pneumococcal, adult Tdap, and RZV vaccines [24]. They pointed out that the RZV vaccine contains the same adjuvant as GSK's RSV vaccine, AS01_E_.

## 4 Discussion

Our review found that adult vaccine coadministration is safe for all the combinations we assessed. Most AEs were generally mild to moderate and of short duration. Some studies showed slightly more reactogenicity with coadministration but few or no serious AEs or safety signals.

Nearly every study confirmed that coadministration had no significant effect on immune response for either vaccine. In the few studies that identified a decreased immune response related to coadministration, the differences were either not clinically relevant or of unknown clinical significance. Some evidence shows that uptake of vaccine coadministration among adults has increased because the ACIP recommended coadministration of COVID‐19 and influenza vaccines in advance of the 2021–2022 influenza season, but the rates remain low.

On the basis of data collected from November 2021 to January 2022, Radner et al. found that coadministration of the influenza vaccine and the Pfizer COVID‐19 booster vaccine decreased the immunogenicity of the COVID‐19 booster vaccine, which they hypothesized could result in more breakthrough infections [6]. On the other hand, McGrath's comparative effectiveness study of a diverse population of more than 3 million US adults who received the Pfizer bivalent vaccine plus an influenza vaccine between August 2022 and January 2023 determined that, in the community setting, the effectiveness of coadministration was similar to that of the COVID‐19–vaccine‐only and influenza‐vaccine‐only groups [11].

The benefits of coadministration are clear. For example, Chen et al. concluded that, in line with WHO recommendations for prenatal vaccination, combined vaccination for COVID‐19, influenza, and Tdap is a safe and feasible strategy to increase protection against these diseases for pregnant women and their newborns [27]. Izikson et al. pointed out that coadministration of COVID‐19 and influenza vaccines could reduce morbidity and mortality [5]. Several authors suggested that adult vaccine coadministration could ease pressure on health systems [5, 12, 19]. Melgar et al. suggested that providers deciding whether to coadminister other vaccines with an RSV vaccine should consider whether the patient is up to date with currently recommended vaccines, the feasibility of the patient returning for additional vaccine doses, the patient's risk for acquiring vaccine‐preventable disease, vaccine reactogenicity profiles, and patient preferences [24].

The benefits of vaccine coadministration outweigh the risks. It increases convenience for vaccinees, which contributes to increased uptake of recommended vaccines in a timely manner. As a result, more people are protected against vaccine‐preventable diseases, including those who cannot tolerate vaccination, thanks to community immunity. Coadministration reduces the number of missed opportunities to keep adults up to date on recommended vaccinations. Avoiding missed opportunities to vaccinate may be particularly important in the adult population due to limited interactions that many adults have with the healthcare system. Finally, because coadministration decreases the number of visits required for vaccination, it contributes to the efficient use of healthcare resources, which can bring down costs of care.

Vaccine coadministration involves few risks. In some cases, vaccine coadministration increases the risk of reactions, but they are generally mild and resolve quickly. Coadministration is contraindicated in certain situations. For example, in persons with anatomic or functional asplenia and/or HIV infection, quadrivalent meningococcal conjugate vaccine and pneumococcal conjugate vaccine should not be administered simultaneously [2]. Finally, there is little evidence that coadministration affects immunogenicity; our review found that a few studies have shown some signals as measured by serology, but the clinical relevance of these findings remains unknown.

Finally, we note that there remain data gaps with regards to the co‐administration of adjuvant‐containing vaccines. In a publication by Schmader et al., the authors looked at the simultaneous administration of an adjuvanted quadrivalent influenza vaccine (aIIV4) and the adjuvanted recombinant herpes zoster vaccine (RZV) in community‐dwelling adults aged 65 years or older and concluded that coadministration was safe and well‐tolerated. The study reported that the percentage of participants experiencing severe reactions was similar between those receiving both vaccines together and those receiving them separately [31].

Phase 3 clinical trial data involving adults aged 50 and older to evaluate the immunogenicity, reactogenicity, and safety of coadministering two adjuvanted vaccines, RSV and RZV, have been presented in an abstract at the 2024 European Geriatric Medicine Society meeting. This study found that the coadministration of the two vaccines elicited a non‐inferior immune response for both vaccines compared with separate administrations. Additionally, the combined vaccination was well‐tolerated, with acceptable reactogenicity and safety profiles [32].

The CDC concludes, “With some exceptions, simultaneously administering the most widely used live and non‐live vaccines has produced seroconversion rates and rates for adverse reactions similar to those observed when the vaccines are administered separately.” [2] The COVID‐19 pandemic highlighted the critical role of vaccination in maintaining individual and public health. Currently, CDC's Advisory Committee on Immunization Practices (ACIP) recommends that all adults receive COVID‐19 vaccination and annual seasonal influenza vaccination, with several other vaccines recommended for most [33]. Now is the time to increase adult immunization rates through coadministration to prevent and mitigate vaccine‐preventable diseases.

## 5 Conclusion

Our review primarily looked at the newest vaccines (COVID‐19 and RSV) in combination with the most common adult vaccine (influenza) and found that coadministration with these and other vaccines in adults is safe, effective, and acceptable. As of June 2024, the ACIP adult immunization schedule includes 15 vaccines for adults, nearly as many as recommended for infants and children [33]. Achieving adequate vaccine coverage among adults will require a coadministration strategy similar to the approach for the pediatric population.

WHO emphasizes that immunization is key to primary healthcare and a reliable investment in human health [34]. CDC and Immunize.org point out that vaccines can prevent infection and spread of diseases that not only affect health but can also result in missed work, medical bills, and inability to meet other obligations, such as caring for other family members [35, 36]. These and other entities across the globe recognize that vaccine coadministration is a feasible approach to reduce the impact of vaccine‐preventable diseases.

Numerous resources are available to help clinicians seeking to incorporate vaccine coadministration in their practice. For example, the Global Influenza Initiative provides helpful infographics about coadministration and related topics [37]. The International Pharmaceutical Federation provides guidance for pharmacists [38]. Immunize.org offers many resources for adult vaccines, including a one‐page reference table on administration [39], another to help determine which vaccines a patient should consider [40], and how to address anxiety around vaccination [41]. We believe that the potential benefits of vaccine coadministration for adults outweigh the slight risks identified. Clinicians have a critical role to play in boosting individual and public health by offering vaccine coadministration.

## Author Contributions

**Litjen (L.J) Tan:** conceptualization, investigation, writing – original draft, methodology, validation, writing – review and editing, data curation, supervision, project administration, formal analysis. **Dana Trevas:** investigation, writing – original draft. **Ann R. Falsey:** conceptualization, investigation, writing – review and editing, methodology.

## Conflicts of Interest

A.R.F. has received grants from Janssen, Merck, CyanVac, VaxCo, BioFire Diagnostics, Moderna, Pfizer, and AstraZeneca. A.R.F. has received consulting fees from ADMA Biologics, GSK, Sanofi, Merck, and Shinogi; support for attending meetings and/or travel from GSK, Moderna, and Sanofi; and has participated in a Data Safety Monitoring Board/Advisory Board for Novavax. L.J.T. and D.T. report no conflicts of interest.

### Peer Review

The peer review history for this article is available at https://www.webofscience.com/api/gateway/wos/peer‐review/10.1111/irv.70090.

## Data Availability

The data that support the findings of this study are available from the corresponding author upon reasonable request.
